# Are the Subjects of Convulsion and of Anesthesia Conscious?

**Published:** 1854-01

**Authors:** F. A. Ramsey


From the Southern Journal.

### *Are the Subjects of Convulsion and of Anesthesia conscious?*

Hypocrates in one of his aphorisms says that a person suffering pain without realizing it, is in very great danger. This assertion of the great observer, should not be permitted to pass without attention or with that smile of complacency with which the remark has been met by some, in whose presence it has been repeated. There may be simplicity in the affirmation, that pain exists without the knowledge or consciousness of the affected individual, but it is believed nevertheless to be an actual fact, by more than a moiety of the correct observers of animal functions, and human sufferings

The question has been discussed, indeed the ground has been assumed, that the individual subject of surgical manipulation and under the influence of ether, chloroform, or any of the analogous agents, is not actually void of pain, or destitute of consciousness of its existence; but like the inebriated man, who acts consciously from the impulse of the moment, yet, when free from the agent which has produced the impression, all recollection—memory—is dead—the same as if the pain had not existed, the act had never been committed.

So too in convulsions, the practitioner has often from the stimulous of his own desire to know, as well as the sympathy or curiosity of by-standers or friends, to speculate as to the positive experience of suffering by his patient. For the most part, from the fact, that after the convulsive seizure has passed off the patient has no knowledge, no rememberance of the circumstances which had given to others so much cause for uneasiness, the question has summarily answered that though rigid and relaxed, spasmed and distorted, contorted and awfully tossed by the convulsive power, yet the patient did not positively suffer—had no knowledge of the agony endured, as evidenced during the continuance of active eclamptive. Such an opinion has been the comfort of the writer, who believes that it has been his misfortune to see more of convulsion than any one of his age in practice, until he was forced to entertain doubt.

The man with his senses bewildered, mind clouded, and sentiments debased by the influence of alcoholic drinks, may receive a castigation or a serious personal or physical wound, and cry out with lamentations and groans, and yet not be able after the poisonous influence has been permitted to die away, to tell when or how it was received—indeed may never know unless informed by others that he has submitted to an indignity, or by the blood and wound that he has met with an accident, or received an injury. And again, these things may occur and the memory, when the individual is first passing into a normal condition, will preserve very faintly the impression made at the moment of occurrence. This has been observed very frequently though possibly not reflected upon by every one in this country, where unfortunately the opportunity is so often presented, by the unwise and illiberal perversions of the use of alcohol.

The same has been observed by every one who has employed the anesthetic agents to any extent. Patients have during operations given every evidence that they suffered all the torment known to be inseparable from the particular operation, and yet, after all has been accomplished, and the mind permitted to resume its throne, have no remembrance of anything during the moments passed under anesthetic influence; while others have a dim recollection of circumstances as they occurred, or of the circumstances somewhat perverted.

Is this not the case with persons under convulsive attacks? A case has presented to the writer's observation, which impressed him with peculiar force.

An interesting boy aged about three years, was observed by his parents to be unwell. Having lost two or three children by very sudden spasms, their fears were active, and the physician was promptly summoned. On examination no wireness or tension of the pulse, or nervous twitchings of the tendons could be discovered and the apprehensions were attempted to be allayed. After a few moments he was observed to bend his head to one side and down towards a shoulder. His mother asked him "what's the matter," to which he replied very faintly, "nothing." This was repeated three times, the same answer given twice, but no notice whatever the third time. But a very few moments elapsed, when he was seized with a most violent and general convulsion, distorting his countenance and affecting the muscles of his whole body and limbs; lasting for full fifteen minutes actively, when it passed off, leaving him in a listless, and apparently unconscious state for full half an hour longer. At the end of this period he opened his eyes, looked round, threw himself his full length, and very languidly exclaimed "what a hard ride that was I had on the wagon." It was indeed a hard ride, in a rough wagon, over a rough road; for I believe, though I have seen them of a longer duration, I have never seen a more violent action than that convulsion; and I am sorry to believe that the little fellow knew his sufferings, during their continuance, though he remembered them but for a few moments immediately after their subsidence.

The case I have thought to be worthy of record as a fact, which may with others, establish a conclusion something like positive, if it will not in itself settle a principle.

Sept. 10, '53. F. A. RAMSEY.

